# The skin microbiome: from historical ecology to therapeutic frontiers^؆^

**DOI:** 10.1016/j.abd.2026.501393

**Published:** 2026-06-27

**Authors:** Sabri Saeed Sanabani

**Affiliations:** Laboratory of Medical Investigation LIM-56/03, Faculty of Medicine, Universidade de São Paulo, São Paulo, SP, Brazil

**Keywords:** Atopic dermatitis, Gastrointestinal microbiome, Infectious skin diseases, Microbial dysbiosis, Skin microbiome, Therapeutics

## Abstract

**Background:**

Human skin, the body's largest organ, hosts a diverse ecosystem of bacteria, fungi, viruses, and mites collectively known as the skin microbiome. This microbiome supports cutaneous homeostasis through barrier defense, immune education, and metabolic functions.

**Objective:**

To narratively review the historical evolution of skin microbiome research, synthesize current knowledge on its composition, biogeography, and functional roles in health and disease, and highlight emerging microbiome-based therapeutic strategies in dermatology.

**Methods:**

This review integrates seminal historical works with contemporary evidence from culture-independent sequencing and multi-omic investigations of the skin microbiome, identified through a selective search of recent dermatology and microbiome literature.

**Results:**

Modern molecular and multi-omic approaches have revealed microbial diversity across sebaceous, moist, and dry skin niches and clarified key functions of the skin microbiome, including colonization resistance, immune modulation, metabolite production, and participation in the gut-skin axis. Dysbiosis of these communities is linked to inflammatory conditions such as atopic dermatitis, acne vulgaris, psoriasis, and chronic wounds. A growing body of work supports microbiome-targeted interventions, including probiotics, prebiotics, postbiotics, and microbiome engineering, as promising personalized strategies.

**Study limitations:**

As a narrative review, this work may be subject to selection bias and does not provide a quantitative synthesis of all available studies on the skin microbiome.

**Conclusions:**

By integrating historical context with mechanistic insights from modern microbiome research, this review underscores the skin microbiome as a central ecological determinant of cutaneous health and disease and provides a framework for translating microbiome science into clinical applications and precision dermatology.

## Introduction

The human body is a complex superorganism, composed of human cells and a large consortium of microorganisms that together make the human microbiome.^1^ The gut microbiome has obviously had the most attention from both a scientific and public health standpoint; however, commensal microorganisms living on our skin are now being shown to be equally important in health and disease.^2^, ^3^ The skin of humans is a large organ (average surface area of nearly two square meters) that serves as the primary interface between the host and the outside environment.^4^ Importantly, the skin is an active ecosystem that is colonized by a populous and diverse array of bacteria, fungi, viruses, and microscopic arthropods.^5^, ^6^ This resident microbiota, which is referred to collectively as the skin microbiome, exists in sophisticated, symbiotic relationships with its host, impacting everything from barrier integrity to immune responses to nutrient turnover.^7^, ^8^, ^9^

The study of the skin microbiome involves microbiology, immunology, dermatology, and ecology. The study of the skin microbiome necessitates a departure from conventional views of the conventional pathogen-centric view of skin disease, which focuses on identifying and eradicating a unique disease-causing organism.^10^, ^11^ The authors now study a skin that is an ecological landscape with distinctive microenvironments, each with specialized physiological properties (e.g., pH, temperature, moisture, and sebum content) that select for varied specialized microbial communities.^12^, ^13^ The ecological concept of homeostasis, or balanced, cooperative interactions of host and microbial residents, is critical to understanding how the microbiome maintains homeostasis.^14^, ^15^

In a state of health, the microbiome of the skin has critical functions.^16^ It physically prevents colonization by opportunistic pathogens through competition and the production of antimicrobial compounds.^10^, ^17^ It is also in a constant dialogue with the immune system of the host, from both the aid of microbiota in educating and configuring immune response by promoting tolerance toward commensal organisms while remaining vigilant against authorized pathogens and invaders.^18^, ^19^ Microorganisms also contribute to metabolomic processes by breaking down lipids and other natural products on the skin surface to generate compounds to benefit the host.^20^

In the opposite direction, perturbations to this delicately balanced state, referred to as dysbiosis, are being recognized as increasingly relevant for the development of a number of skin diseases.^21^, ^22^ The inflammatory skin diseases of Atopic Dermatitis (AD), acne vulgaris, psoriasis, and rosacea, in addition to failure of wound healing, are all recognized to have distinctive changes associated with the skin microbiome composition and function.^23^, ^24^ Understanding how or what triggers the shift from homeostasis to dysbiosis is an area of active research that is hoped to return innovative diagnostic tools and treatment methods.

This review serves to combine the historical context of the skin microbiome with the emerging scientific understanding. The authors will begin by examining the history of skin microbiology, beginning with the initial findings, through advancements in technology that have allowed for exploration of the microbial world in some depth. Next, the authors will define the expected composition and surface topography of the skin ecosystem, identify the functions of the microbiome in immunity and health, and make connections with dysbiosis and common skin diseases. Lastly, the authors will end with a discussion of the research and new therapeutic horizons that are about to change dermatology, including interventions that contain the microbiome, as well as a future of personalized skin products based on our unique type of microbes that inhabit our skin. This pathway through ecology to emerging therapeutic perspectives highlights the importance of microorganisms shared with the skin, thus repurposing this essential state from passengers to partners in human health.

## Historical perspectives: The Genesis of skin ecology

The understanding of the skin as a microbial ecosystem is a fairly new awareness on the grand scale of history, but long-forgotten history considers the roots of this perspective in the field of microbiology. From viewing the “animalcules” on the skin, to mapping these organisms into communities with functional roles, maps the large evolution of thinking and technology, which ultimately displays an evolution from the reductionist views of pathogenic microorganisms to a prominent ecological role of microbiota resident flora in human physiology. Importantly, this was not accomplished in years, but in decades of pioneering discoveries in purposeful concepts, approaches, and technologies.

### Early discoveries in cutaneous microbiology

Our journey into the wonder of microbes began in the 17^th^ century, when Antonie van Leeuwenhoek devised the microscopic lenses that allowed him to observe microorganisms and record their findings based on samples taken from his own skin.^25^ However, the systematic study of the little creatures started later in the 19^th^ century during the golden age of microbiology with Louis Pasteur and Robert Koch, who were primarily responsible for understanding germ theory of disease and proving the pathogenic relationship with specific illnesses and diseases.^26^, ^27^ The focus of this time was centered around the establishment of isolation approaches and the development of the first agar-based media (that could grow bacteria) lead to the concept of identifying bacteria in pure culture of “isolations”.

Some of the first approaches to discover what microorganisms are involved with skin infections were made during this period of culture. In the 1880s, Alexander Ogston examined pus from human abscesses and identified clustered micrococci as the cause of suppuration, coining the term *Staphylococcus* from the Greek for ‘bunch of grapes’. These organisms, including *S. aureus* and *S. epidermidis*, are now recognized both as common members of the human skin microbiota and as important causes of skin and wound infections.^28^, ^29^ Paul Gerson Unna, as the first dermatopathologist who interpreted bacteria with respect to skin conditions, also provided groundwork by suggesting *Pityrosporum ovale* (now *Malassezia*) was a contributing factor in the appearance of seborrheic dermatitis.^30^

While these advancements were achieved, they were limited by the cultivation-dependent approaches used. The methods were limited to the organisms that could grow in laboratory conditions (typically fast-growing, aerobic, and resistant bacteria). As a result, the vast number of organisms that were anaerobic, slow-growing, or had complex nutritional needs were never examined, thereby biasing the findings by providing an incomplete view of the skin's microorganisms. This “great plate count anomaly”, where the organisms observed with a microscope outnumbered those that could be cultured, suggested a multitude of unobserved organisms were waiting to be characterized. Overall, anyone studying skin flora at that time had a simplistic view that these few, culturable microbes collectively made up the skin flora and were only important if they caused disease.

### Major contributions of Mary Marples: Ecology paradigm shift

A sea change in the human skin microbiome happened as microbial ecologist Mary Marples from New Zealand revolutionized how the authors think about cutaneous microbes in the 1960s. Marples published “The Ecology of the Human Skin”, in 1965, which was an influential text that fundamentally shifted the view of cutaneous microorganisms. Marples introduced the notion that human skin should no longer be viewed as a simple surface but instead a habitat, examining it through the lens of other ecologically characterized systems, i.e., plant and animal ecology.^31^

Marples went beyond just listing organisms to understanding the distribution and rules of interactions that led to their distributions. She provided a specific characterization of the topography of the skin, grouping regions into analogous geographical biomes that included the “axillary jungle”, “forehead plain”, and “forearm desert”. She then began to address how factors of the host, such as moisture, pH, temperature, and nutrition (mostly from sebum), rested selectively on both the host and the microbes’ organismal distributions.^31^ Most notably, she changed the perception of the host-organismal relationships to include a full spectrum from commensalism to mutualism and parasitism rather than antagonistic thinking, allowing for the entire skin to be thought of as an ecosystem.

Her work suggested that the resident microbiota was stable and well-adapted to the environment, and therefore resistant to invasion by transient or pathogenic organisms. This idea led to a new understanding of the idea of “colonization resistance”, the most protective function of a healthy microbiome. Marples was able to pivot the importance of interactions of host and microbes to explain health and disease states; utilizing ecology as the foundational concept for this thinking was a bold move in time.^32^ Most importantly, she shifted the thinking from “What pathogens are present?” to the question of “What is going on in the ecology of the entire microbial community and how does it affect the host?” Marples made substantial contributions to an ecological view of the skin that have persisted to this day and served as a precursor to the molecular revolution that followed.^31^

### Evolution of methods: culture to code

The full potential of Marples's vision could not be realized due to the tools available at the time. The ecology of the skin and all potential organisms remained hidden until the use of culture-independent molecular tools developed in the late 20^th^ and early 21^st^ century. These new approaches changed the game for researchers. Transitioning from cultivation to sequencing approaches, researchers could minimize bias in our understanding, analyze whole communities of organisms, and directly analyze their genetic material.^33^

The first major breakthrough was the use of 16S ribosomal RNA (rRNA) gene sequencing. The 16S rRNA gene is an ideal molecular marker, as it is universally present in all bacteria and archaea, and contains highly conserved regions (for designing universal primers) and variable regions (which provide a “barcode” for taxonomic identification).^34^, ^35^ In this way, researchers could sequence the 16S rRNA gene directly from a skin sample, and generate a census of the bacterial skin community to identify hundreds ‒ sometimes thousands of species, most of which had never been cultured in a laboratory.^36^ This degree of skin bacterial diversity was previously undreamed of and that microbial communities systematically differed at various skin sites.

With the advent of Next-Generation Sequencing (NGS) technologies, DNA sequencing throughput increased with costs declining, allowing for large-scale projects (e.g., Human Microbiome Project).^37^ This significant step forward initiated an explosion of work characterizing the skin microbiome in health and disease. For example, the authors have transitioned from simply asking “who is there” (taxonomic profiling from 16S rRNA sequencing), to start to ask, “what are they doing” (amount of functions).

**Fig. 1:**
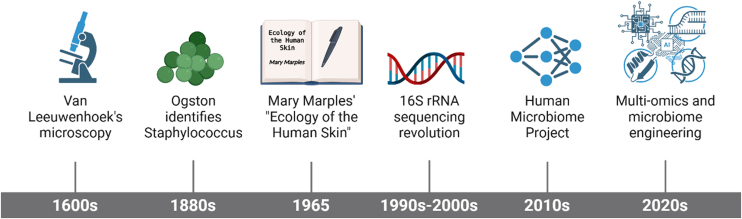
**Chronological evolution of skin microbiome research**. The timeline highlights key milestones from the 17th-century discovery of “animalcules” by Van Leeuwenhoek to the 1965 ecological paradigm shift by Mary Marples, leading to the modern era of 16S rRNA sequencing, the Human Microbiome Project, and current multi-omics approaches. Created in https://BioRender.com.

Shotgun metagenomic sequencing ‒ sequencing all genomic DNA in a sample ‒ is the comprehensive technique for understanding the total metabolic potential of the community, i.e., identifying all the genes. With recent advances, the authors are moving toward multi-omic approaches that combine metagenomics with other high-throughput methodologies. For example, metatranscriptomics (RNA) tells us which genes are being expressed, metaproteomics (proteins) identifies the functional machinery of the cells, and metabolomics (small-molecule metabolites) captures the biochemical output of the community. The advancement of these high-resolution technologies is beginning to advance our understanding of the functional dynamics of the skin microbiome and their complex interactions within a host.^38^ The transition from Petri dish work to high-performance computing has revealed the skin as the complex, dynamic, and important ecosystem that Mary Marples envisioned. This transition from culture-dependent to molecular methods has finally realized the comprehensive ecological vision that Mary Marples articulated decades earlier, revealing the skin as the complex, dynamic, and functionally important ecosystem she envisioned, while opening new opportunities for therapeutic interventions, including the rapidly evolving field of skin microbiome engineering^39^ (Fig. 1).

## The Skin as an ecosystem: Composition and topography

Building upon the ecological framework established by pioneers like Mary Marples, modern molecular techniques have revealed the skin's microbial landscape in unprecedented detail. This section examines the key microbial inhabitants colonizing human skin, their spatial distribution patterns across anatomical sites, and the complex interplay of environmental factors that shape these site-specific communities. Rather than viewing the skin as a uniform surface, the authors now recognize it as a collection of distinct microenvironments, each with unique physiological characteristics that select for specialized microbial assemblages.^40^

### Key microbial inhabitants: bacteria, fungi, viruses, and mites

The skin microbiome can be considered a multispecies consortium, in which members of the bacterial kingdom are the most ubiquitous and studied. The skin microbiome is primarily represented by four major bacterial phyla: Actinobacteria, Firmicutes, Proteobacteria, and Bacteroidetes. At the genus level, some of the dominant bacteria include *Cutibacterium* (formerly *Propionibacterium*), *Staphylococcus*, and *Corynebacterium*. *Cutibacterium acnes*, an anaerobic lipophilic bacterium, predominates in sebaceous follicles of the face and back, where it has evolved specialized metabolic capabilities to thrive in lipid-rich environments.^5^, ^41^ Some *Staphylococcus* are commensal occupants of exposed dry surfaces, such as the antecubital fossa (e.g., *S. epidermidis*). Other genera include *Corynebacterium*, which prefer moist areas, such as the axilla (i.e., armpit) or groin, and are thought to contribute to body odor production.^42^

In addition to the bacterial kingdom, the skin microbiome also has an abundant fungal community that is referred to as the skin mycobiome.^36^ Although representing a smaller proportion of the total microbial biomass when compared to bacteria, fungi are nevertheless key players in skin health and disease.^43^ The most dominant genus of fungi, representing the skin mycobiome, is *Malassezia*, a lipophilic yeast that normally inhabits seborrheic (i.e., oily) skin sites on the scalp, face, and chest and is thought to be responsible for dandruff and seborrheic dermatitis during dysregulated population growth.^36^ Other genera of fungi, such as *Candida*, *Aspergillus*, and *Cryptococcus*, have been identified, but are usually in smaller relative abundance, and can opportunistically act as pathogenic organisms when the skin dysregulates.^44^, ^45^

The viral component of the skin microbiome, or skin virome, represents the least understood member of the skin microbiome, but is becoming an area of increased interest. The skin virome is primarily comprised of bacteriophages (i.e., viruses of bacteria), which likely perform some significant role in regulating bacterial abundance with predator-prey interactions and can mediate the overall structure and stability of bacterial communities.^46^, ^47^, ^48^ For instance, phages targeting the key bacteria of *C. acnes* or *S. aureus*, could conceivably shift the population density of the corresponding bacteria with some consequence for conditions like acne and AD. Other components of viruses that are eukaryotic in origin and are grouped in the skin virome include human papillomaviruses (HPVs) and polyomaviruses.^49^, ^50^ While some HPVs are the cause of warts and skin cancer, many HPVs are carried asymptomatically by healthy subjects, indicating a far more complicated, potentially commensal relationship with the bacteria.^49^, ^51^

Finally, skin also has a diverse ecosystem of microscopic arthropods; most notably, *Demodex* mites. These eight-legged mites are represented by *Demodex folliculorum* and *Demodex brevis*, and they are commonly found in hair follicles and sebaceous glands of humans, including the face and other body areas.^52^, ^53^ For the majority of people that harbor them, *Demodex* are innocuous. However, overgrowth of *Demodex* mites has been linked with inflammatory skin diseases, such as rosacea and blepharitis.^54^, ^55^ This demonstrates that *Demodex* mites exhibit a range of interactions with human health, and they may shift from commensals to pathogens by triggering an inflammatory response or serving as transporters of pathogenic bacteria.^56^ The dynamic interactions among these microorganisms and metazoans ‒ bacteria, fungi, viruses, and mites ‒ create a heterogeneous, complex ecological network that is essential to normal skin function.^5^, ^57^

### Biogeography of the skin: site-specific microbial communities

**Fig. 2:**
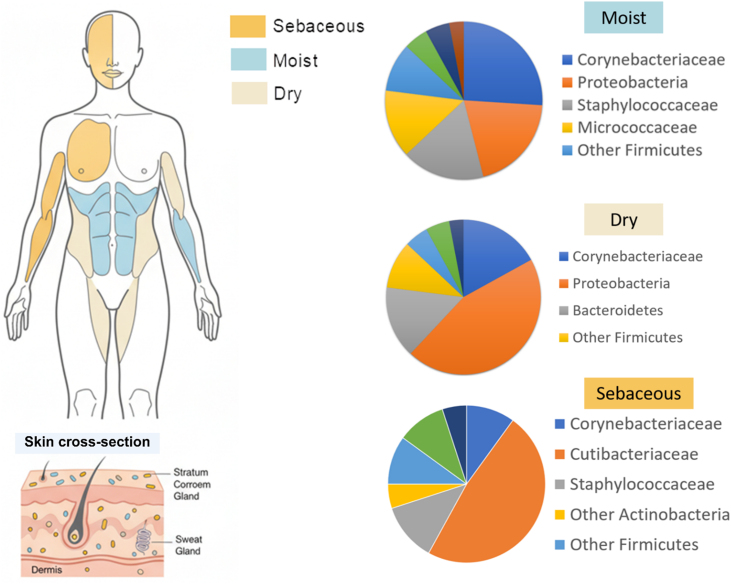
**Biogeographical distribution of skin microbial communities.** (Left) Mapping of sebaceous, moist, and dry anatomical niches. (Right) Pie charts showing the characteristic bacterial composition at the family and phylum levels for each environment. Note: Although bacterial families dominate the biomass, these niches also contain a diverse mycobiome (mainly *Malassezia*) and microeukaryotes such as *Demodex* mites, which are part of the broader ecological network . Created in https://BioRender.com.

The human skin is not a flat surface; it is a vast landscape of different microenvironments with distinct physiological properties. Physiological and topographical heterogeneity give rise to an extraordinary pattern of microbial biogeography, with new microbial communities assembling at different body sites.^58^, ^59^ There are mainly three categories of skin sites that relate to the local environment: sebaceous (oily), moist, and dry.^5^, ^60^ These ecologically distinct sites host specific and specialized microbial communities that have adapted to the local environment, similar to climate types on Earth, such as forests and deserts^61^ (Fig. 2).

Sebaceous sites include the forehead, nasolabial folds, chest, and back, and are rich in lipids produced by the sebaceous gland. These oily environments are anaerobic and lipophilic microorganisms preferentially grow in these sites. The dominant microbiota is bacteria of the genus *Cutibacterium*, particularly *C. acnes*, which have enzymes (lipases) that transform sebum triglycerides into free fatty acids for energy.^62^, ^63^ These fatty acids also contribute to the acidic pH of skin, which prevents pathogenic microbial growth. *Malassezia* is a genus of fungi that thrives in lipid-rich areas of sebaceous sites and is considered a core member of the mycobiome of sebaceous sites.^64^, ^65^

Moist sites include the axillary vault (armpit), antecubital fossa (inner elbow), popliteal fossa (behind the knee), groin, and plantar surface (sole of the foot). These areas of skin contain higher humidity and temperature due to skin-on-skin occlusion. Warm, humid conditions provide a perfect environment for bacteria to thrive in aqueous environments. Thus, moist sites of skin are primarily colonized by *Corynebacterium* and *Staphylococcus species*. The metabolic activity of *Corynebacterium* species in the axilla plays the primary role in breaking down odorless precursors (e.g., glutamine conjugates, cysteine-glycine conjugates) from apocrine sweat into volatile organic compounds such as branched-chain fatty acids (e.g., 3-methyl-2-hexenoic acid) and thioalcohols, which produce most axillary body odor; however, *Staphylococcus* (e.g., *S. hominis*) and other taxa contribute shorter-chain fatty acids and additional malodors.^66^, ^67^

### Environmental factors shaping skin biogeography

The distinct microbial communities inhabiting sebaceous, moist, and dry skin sites do not arise by chance, but rather reflect sophisticated ecological filtering processes driven by local environmental conditions. Understanding the mechanistic drivers of this biogeographical patterning requires examining both abiotic factors ‒ the physical and chemical properties of skin microenvironments- and biotic factors ‒ the competitive, cooperative, and antagonistic interactions among community members. These factors do not operate in isolation; rather, they create complex selective pressures that determine which microorganisms can successfully colonize, persist, and function at specific anatomical locations. The following sections detail the key abiotic and biotic determinants that sculpt the skin's microbial landscape, ultimately linking environmental heterogeneity to community structure and function.

Abiotic factors represent the non-living environmental characteristics that impose primary constraints on microbial community assembly:

• Temperature: Skin surface temperatures vary throughout the body; odors in warm, occluded areas such as the axilla and groin are associated with a different microbiome than cooler, less occluded sites such as the forearm.
• Moisture: Moisture levels vary widely at dry, moist, and oily sites. Water availability is a basic requirement for microbial growth and is a primary driver of the biogeographical patterns observed on the skin.
• Sebum: The lipids produced by sebaceous glands provide a nutrient source for many lipophilic microbes such as *Cutibacterium* and *Malassezia*, and sebum levels are consequential in determining the community composition of oily areas.^60^, ^68^
• Host genetics and immune status: Host genetics modulate epidermal barrier integrity and local immune responses, which in turn influence the level and repertoire of skin antimicrobial peptides (AMPs) and thereby exert selective pressure on the cutaneous microbiome.^69^, ^70^
• External factors: Lifestyle choices and environmental exposures significantly influence the skin microbiome. Cosmetics, soaps, and moisturizers can increase skin surface pH and alter lipid classes, favoring pathogenic shifts; most skin medications, especially antibiotics, cause more profound and long-lasting microbiome disruptions with slow recovery, and diet, occupation, and geographical location also contribute unique microbial signatures.^5^, ^71^, ^72^

Biotic factors are the interactions between the living organisms in the ecosystem. The skin is a crowded environment where microbes constantly compete for growth opportunities in space and resource use, which drives sophisticated inter-species and even intra-species dynamics:

• Competition: Microorganisms compete for limited nutrients and available attachment sites on the skin surface. Commensal bacteria such as *S. epidermidis* can outcompete and displace pathogens such as *S. aureus*, a process termed colonization resistance.
• Antagonism: Many resident microbes produce antimicrobial substances directed towards inhibiting or killing competing organisms. For example, *S. epidermidis* secretes bacteriocins and AMPs that are specifically active against several strains of *S. aureus*.^73^, ^74^
• Metabolic cooperation: Microbes engage in cooperative interactions through metabolic cross-feeding, in which certain species break down complex molecules from the host ecosystem (e.g., triglycerides by *C. acnes*) into simpler by-products (e.g., free fatty acids) that serve as nutrients for other community members, such as *Malassezia restricta*.^75^
• *Quorum* sensing: *Quorum* sensing enables bacteria to communicate via autoinducers, synchronizing gene expression based on population density to coordinate behaviors such as biofilm formation and virulence factor production, thereby influencing community structure and functionality.^76^
• Bacteriophage predation: Bacteriophages exert top-down control over bacterial community structure by selectively lysing certain bacteria while sparing others, thereby maintaining overall compositional diversity; this prevents any single species from dominating and shapes microbial ecology.^77^

Abiotic and biotic features are not separate; they are intertwined.^69^ Host abiotic features create an environmental filter in the skin ecosystem that determines which microbes are able to establish a foothold in the population. The resulting biotic interactions, or relationships between native community members, further refine local community composition and function, and this balance is required for ecological stability and a healthy skin microbiome.

## Functional roles of the skin microbiome in health

The resident microorganisms of the skin are not simply passive, incidental residents, but also active participants in skin homeostasis. This relationship (of established human symbiosis) has taken millennia to become optimized among the multitude of resident microbes and the host. The skin microbiome provides a suite of beneficial functions, from acting as a first line of defense against pathogenic species to educating and calibrating host immune function. Metabolically, these organisms contribute to nutrient processing and to maintain the physicochemical characteristics of the skin. Furthermore, there is developing evidence that the microbial communities of the gut and skin are fundamentally related, which demonstrates a system for 'crosstalk' in which organisms communicate between different organs to promote skin health.^78^, ^79^ Knowing about these functional roles is additionally necessary to understand why the functionality of the microbiome is important to the overall health of the skin.

### Barrier function and protection against pathogens

Among its many functions, the skin microbiome provides the skin with a barrier defense system. The protective role assumed by skin microbiota can be discussed through a variety of different co-dependent functions, which, together, provide a large barrier to the colonization and invasion of pathogenic microorganisms, and can be described as follows:

First, the commensal microbiome provides colonization resistance. This is an important concept in some areas of microbial ecology. The resident microbiome occupies physical niches at the surface of the skin, which prevents pathogenic microbes from establishing a foothold. This can be derived from an extremely simple but effective method of competitive exclusion, whereby the presence of a harmless commensal microbe limits the availability of space and resources for a non-resident pathogen. For example, the dense and diverse communities formed by *S. epidermidis* on the epidermal surface make it more difficult for potentially pathogenic transients, such as *S. aureus*, to be stably established in the microbiome.^80^, ^81^

Second, resident microbes directly antagonize incoming pathogens through secreted antimicrobial compounds. This represents a biochemical defense layer distinct from spatial competition. Commensal bacteria produce diverse antimicrobial peptides (AMPs), bacteriocins, and antimicrobial lipids that selectively target potential pathogens.^82^, ^83^ *S. epidermidis* exemplifies this strategy, secreting multiple bacteriocins, including epidermin, which forms pores in target bacterial membranes with specificity determined by bacteriocin composition. Additionally, *S. epidermidis* produces phenol-soluble modulins (PSMs) with potent activity against *S. aureus* and Group A *Streptococcus*.^82^, ^83^ Similarly, *C. acnes* secretes propionic acid, a short-chain fatty acid (SCFA) that both maintains acidic skin pH and exerts direct antimicrobial effects against diverse pathogens.^84^, ^85^

Third, the skin microbiome serves to re-establish the physical and chemical barrier of the skin. The metabolic byproducts of commensal bacteria are critical to maintaining the acid mantle. The metabolism of sebum lipids by *C. acnes* into free fatty acids, and the fermentation of glycerol to SCFAs by *staphylococci* result in off-skinned acidity (pH < 5.0), which is unfavorable for the growth of many pathogens, particularly *S. aureus*, which prefer a more neutral Ph.^64^, ^86^ A healthy acidic skin surface is, therefore, a tangible functional output of the resident microbial community. Additionally, some evidence suggests that microbial signals can enhance the epithelial barrier integrity by eliciting the production of tight junction proteins in keratinocytes, which strengthen the connections between skin cells and reduce permeability.^87^ While these direct antimicrobial strategies provide immediate protection, commensal microbes also engage in more sophisticated immune-educating functions that the authors explore in the following section.

### Education and modulation of host immune system

The skin microbiome also plays a role in developing, educating, and modulating the immune system of the host.^5^ From birth, exposure to commensal microorganisms is critical to teach the immune cells of the skin to differentiate and determine if a signal is harmless resident microbes or pathogens deserving of an immune attack. This event is called immune tolerance, meaning that the immune system is not elicit inappropriate (and damaging) inflammatory attacks on beneficial microbiota.^88^, ^89^

The conversations between microbes and the immune system begin, at least, on the cellular level. Skin cells, including keratinocytes, and amongst resident immune cells such as Langerhans cells and dendritic cells, possess pattern recognition receptors (PRRs), such as toll-like receptors (TLRs) that detect conserved microbial structures known as microbe-associated molecular patterns (MAMPs).^90^ For example, lipoteichoic acid (LTA) is located on the cell walls of Gram-positive bacterium, such as *S. epidermidis*. When the commensal MAMP binds to the PRR on the host cell, it induces signaling events that are important for the calibration of the immune response.^91^

Beyond their direct antimicrobial activities, commensal microbes orchestrate host immune responses to create a second layer of protection. *S. epidermidis* exemplifies this immune-educating function through multiple mechanisms. Specific strains stimulate keratinocytes to upregulate AMP production, essentially recruiting the host's own defensive molecules to reinforce barrier protection.^73^ More remarkably, *S. epidermidis* lipoteichoic acid (LTA) activates TLR2 on keratinocytes, triggering a calibrated response that enhances surveillance while simultaneously dampening excessive inflammatory signaling pathways that would otherwise lead to tissue-damaging cytokine production.^92^ This immune modulation prevents chronic inflammation while maintaining pathogen vigilance.

Furthermore, the skin microbiome can program the adaptive immune response. Commensal microbes have been documented to manipulate the functions of resident T cells in the skin. Various molecules produced by *S. epidermidis* have been reported to serve as stimulators of T cells that can assist in the differentiation of T cells specific for pathogens, such as IL-17-producing T-helper 17 (Th17) cells that are critical for defense against pathogenic fungi and bacteria, such as *Candida albicans* and *S. aureus*.^73^, ^93^, ^94^ Simultaneously, the microbiome stimulates the function of regulatory T-cells (Tregs), which are immune cells specialized for dampening excess inflammatory responses and tolerating commensals.^93^ Therefore, having this balance of T-cells that promote pathogen-specific defenses and Tregs inhabits a healthy host-microbiome relationship. Disruption of the educational conversation, which is present during immune responses, is a key factor in the development of inflammatory diseases of the skin, such as atopic dermatitis and psoriasis, whereby immune responses become dysregulated.

### Metabolically active and natural product degradation

The skin microbiome is a metabolically active community that contributes significantly to the chemical milieu of the skin surface. Resident microbes can produce a vast diversity of enzymes, many of which exceed the host genomic capacity to produce equivalents, to degrade host-derived molecules and environmental compounds that would otherwise be inert.^7^, ^95^ Metabolic activities by resident microbes are also critical for skin homeostasis.

One of the crucial metabolic functions is the metabolism of skin surface lipids.^96^ Lipophilic organisms such as *C. acnes* and *Malassezia*species express lipases that hydrolyze sebaceous triglycerides into glycerol and free fatty acids.^97^, ^98^ This metabolic process serves multiple functions: *C. acnes* utilizes the resulting free fatty acids as an energy source, while *staphylococci* ferment the liberated glycerol into short-chain fatty acids (SCFAs). The free fatty acids contribute to maintaining the skin's acidic pH (typically < 5.0), which inhibits pathogen growth. Triglycerides in sebum can be hydrolyzed into glycerol and free fatty acids via a lipase. Glycerol can be fermented by *staphylococci* into SCFAs, whilst free fatty acids serve as a nutrient source and contribute to the acidic pH the skin maintains; some of these fatty acids, including sapienic acid, also have antimicrobial properties.^99^, ^100^ This metabolic network of bacteria is converting host-secreted sebum into a chemically active, protective layer in the skin.

Skin microbes can also metabolize other host-derived molecules apart from lipids. For example, keratin and amino acids from sloughing corneocytes and sweat are sources of protein that can be potentially degraded. In particular, some skin bacteria can break down arginine, which will produce ammonia that can locally modulate pH.^9^ Commensal microbes also play a role in converting nitrate in sweat to Nitric Oxide (NO), which has potent antimicrobial activity against a wide range of pathogens, significant roles in vasodilation, and wound healing.^101^

Furthermore, the skin microbiome can metabolize xenobiotics, which are organic compounds that originate outside of the body from personal care products, pollutants, or topical medications.^102^ The microbial metabolism generated by the microbiome can either detoxify chemical substances that could be harmful or even activate inert compounds. The so-called “microbial metabolism” of topical products is an emerging area of research that is predicted to have a significant impact on dermatology and cosmetology.^103^ The ability of the microbiome to modify the chemical landscape of skin highlights its importance as a functional partner that is actively shaping the attributes of its habitat–usually in ways that benefit the host.

### The Gut-Skin axis: A systemic connection

While local skin microbiome effects are a well-recognized component of skin health, a growing area of research is the gut-skin axis, a bidirectional communication network between the health of the gut and its microbiome and the health of the skin.^79^ If microbes have some influence on skin homeostasis, that influence is also likely to be systemically modulated by the microbial community of the gastrointestinal tract.

Communication along this axis is likely accomplished by the bloodstream and the immune system. The gut microbiome provides systemic immune education. Gut dysbiosis, or disruption in the gut microbial community, can result in an impaired intestinal barrier (“leaky gut”).^104^ Bacterial components, such as lipopolysaccharide (LPS) from Gram-negative bacteria, as well as microbial metabolites, can enter circulation.^105^ Upon bloodstream entry, these molecules can travel to the skin, where they can stimulate systemic, low-grade inflammation, exacerbating or initiating inflammatory skin conditions such as atopic dermatitis, psoriasis, acne, and rosacea.^106^

The gut microbial community metabolizes dietary fibers into SCFAs, including butyrate, propionate, and acetate, which function as key messengers of this axis.^107^ SCFAs have potent systemic anti-inflammatory and immunomodulating effects.^108^ SCFAs can enter circulation, where they can influence immune cell function throughout the body, including skin, as much of their function is to stimulate the action of anti-inflammatory Treg cells and decrease inflammation overall. Not taking in sufficient dietary fiber risks reduced SCFA generation, which counteracts the systemic anti-inflammatory signal from the gut microbiome and could contribute to a pro-inflammatory state that was manifested at the skin.

**Fig. 3:**
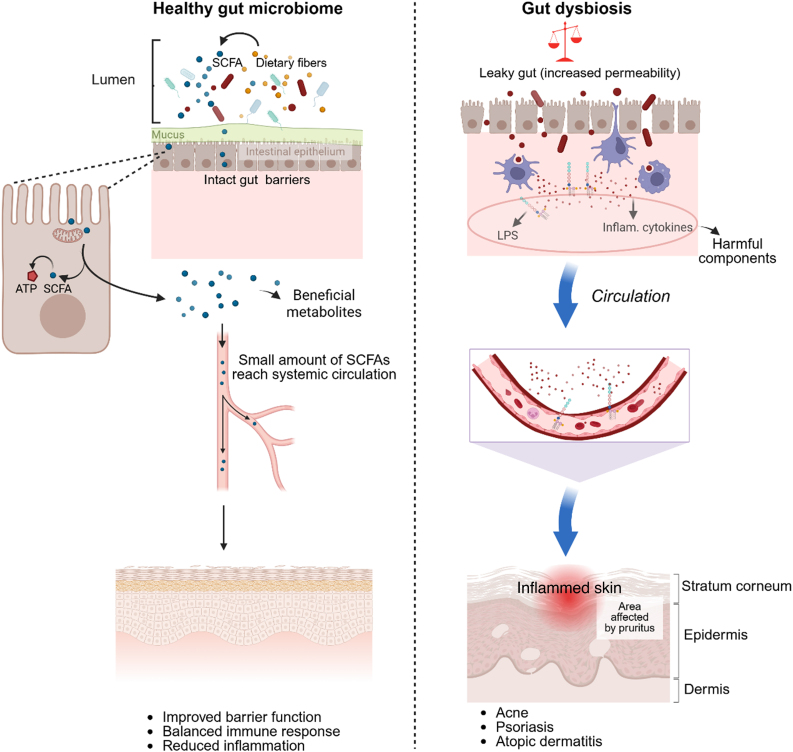
**The gut-skin axis and systemic health.** Comparative overview of a healthy gut microbiome, which produces beneficial metabolites such as SCFAs that support skin barrier function, versus gut dysbiosis. In gut dysbiosis, a “leaky gut” allows translocation of LPS and inflammatory cytokines into circulation, worsening inflammatory skin conditions such as acne and psoriasis. Created in https://BioRender.com.

Clinical and experimental evidence support this association (Fig. 3). Patients with inflammatory skin disease frequently have gut dysbiosis. A similar condition is observed where patients with rosacea have a significantly higher rate of small intestinal bacterial overgrowth (SIBO).^109^ Related to this, several studies have demonstrated alterations in the gut microbiome found in patients with atopic dermatitis and psoriasis.^110^, ^111^, ^112^ Much like asthma, the clinical evidence is primarily correlational; however, oral probiotics (live beneficial bacteria) have provided intervention studies demonstrating improvement in skin conditions such as eczema, thus this supports the “gut-skin axis”.^113^ Overall, this systemic association illustrates how the microbiome can operate as a holistic system, as maintaining the skin barrier requires a balance of local and longer-term factors, including internal factors (e.g. gut health).

## Dysbiosis: The role of The microbiome in skin disease

The fragile balance of the skin microbiome is critical to skin health. When this balance is disrupted, termed dysbiosis, the relationship between the host and microbe can shift from a mutually beneficial relationship to a pathogenic one, contributing to the onset and exacerbation of many skin diseases. Dysbiosis is not merely the presence of a pathogen but rather a broader ecological shift characterized by changes in microbial diversity, the relative abundance of specific taxa, and overall metabolic profile.^114^ In this section, the authors discuss the concept of cutaneous dysbiosis and review its well-characterized roles in some of the most common inflammatory and chronic skin diseases.

### Contextualizing dysbiosis in the cutaneous setting

Cutaneous dysbiosis is considered a functional and compositional maladaptation of the resident microbial population. As compared with the gut microbiome, which is typically characterized by high diversity being a hallmark of health, the “healthy” skin microbiome is highly variable depending on the anatomical niche.^106^ Therefore, cutaneous dysbiosis is contextual and can take several forms: loss of beneficial commensal species, overgrowth of pathobionts (commensal organisms that have pathogenic capabilities), or a general decrease in microbial diversity. These components can paradoxically reduce barrier function, skew the immune response toward inflammation, and create a permissive environment for disease. The shift from a homeostatic to dysbiotic state is typically linked to a combination of host genetics, environmental exposure, and lifestyle factors, highlighting the complexity of the etiology of microbiome-associated skin diseases.^95^

### Atopic dermatitis and microbial imbalance

**Fig. 4:**
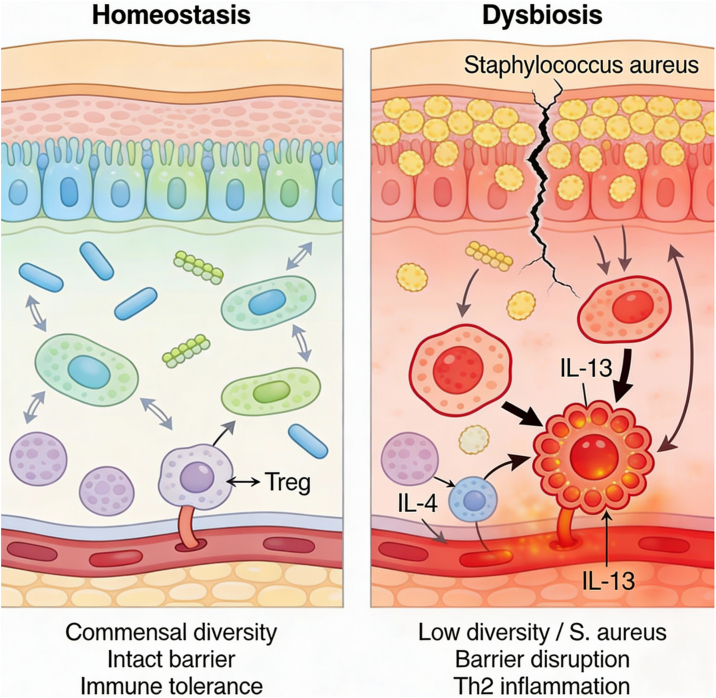
**Mechanistic transition from cutaneous homeostasis to dysbiosis.** Under homeostatic conditions (left), high commensal diversity maintains an intact barrier and immune tolerance. In dysbiosis (right), typical of atopic dermatitis, colonization by *Staphylococcus aureus* leads to barrier disruption and activation of Th2-mediated inflammatory pathways (IL-4, IL-13).

Atopic dermatitis (AD), a chronic inflammatory skin disease, is one of the most studied forms of cutaneous dysbiosis. The skin of AD sufferers typically has a striking decrease in microbial diversity and a pretty dramatic increase in *S. aureus*.^115^ In healthy skin, commensal *S. epidermidis* provides colonization resistance against *S. aureus* through multiple mechanisms, including antimicrobial peptide production and niche occupation.^116^ In flaring AD skin, this protective mechanism is compromised. The growth of *S. aureus* is not simply a by-product, but it is a contributing factor to the disease. *S. aureus* produces toxins and proteases that degrade the skin barrier, increase transepidermal water loss, and directly upregulate type 2 inflammatory pathways. This process creates a cycle of barrier degradation, inflammation, and dysbiosis^117^, ^118^ (Fig. 4). Research into the exact relationship between microbiome shifts and the development of AD is ongoing, but there is experimental evidence that microbiome-based products can restore barrier function and reduce *S. aureus* colonization both clinically and potentially on the skin microbiome.^119^, ^120^

### Acne vulgaris: The complicated role of *Cutibacterium acnes*

Acne vulgaris, a disorder of the pilosebaceous unit, provides a more complex example of dysbiosis. For many years, the generation of acne was thought to be due an over-proliferation of a single bacterium, *Propionibacterium acnes* (now classified as *C. acnes*). However, more recently, several studies employing high-throughput sequencing methods have shown that when *C. acnes* is considered altogether, the relative abundance is often similar between acne-prone skin and healthy skin.^121^, ^122^ Instead, the authors see that dysbiosis/alteration is occurring at the strain level. Several specific phylotypes have been strongly associated with acne lesions, including phylotype IA1.^63^ Strains of *C. acnes* belonging to this phylotype may be termed “pro-inflammatory” because they possess virulence factors that elicit a host inflammatory response while in the follicle. Some other strains of *C. acnes* (often dominant after antibiotics and on healthy skin) seemed to have more of a protective role, rather than inflammatory. Thus, the dysbiosis occurring in this condition is likely better framed as strain-level dysbiosis, not a species-level dysbiotic overgrowth of a single bacterium.^123^ From 2000 to 2023, the acne microbiome literature has expanded steadily, with increasing emphasis on dissecting strain-level pathogenic mechanisms and microbiome‑conserving strategies that target virulent C*. acnes* clades or biofilms while preserving commensal taxa.^124^, ^125^, ^126^

### Chronic wounds and diabetic foot ulcers

The authors can also see the dual role of the microbiome in wound healing.^23^ The microbiome can assist in the repair of tissues when healthy; however, when dysbiotic, it can lead to chronic (non-healing) wounds like diabetic foot ulcers (DFUs). Rather than a single pathogen usually implying lack of healing, typically chronic non-healing (hypofunctional) wounds become colonized by multiple (often complex) polymicrobial biofilm communities, which all contribute to the dysbiotic substrate. Typical dysbiotic characteristics include increased total bacterial burden, higher anaerobic diversity, and the presence of members from the genera *Staphylococcus* and *Pseudomonas*.^127^ These biofilm organisms have the propensity to generate a pro-inflammatory micro-environment, which affects epithelial migration, angiogenesis, and overall wound healing. The advent of molecular biology technologies has also advanced the study of the skin microbiota associated with DFUs; more recent studies have identified composition and metabolic activity of the microbial community as leading determinants of healing outcomes.^128^ Identifying proper dysbiotic signatures for chronic wounds may offer a basis for developing novel diagnostic and therapeutic approaches to achieve successful wound healing, including biofilm-disruptive agents and targeted antimicrobial interventions.

### Psoriasis: Chronic inflammation and the Th17/IL-17 signature

Psoriasis is a systemic, immune-mediated disease characterized by well-demarcated scaly plaques and hyperproliferation of keratinocytes.^129^ Unlike AD, where a single species (*S. aureus*) often dominates, the psoriatic skin microbiome is defined by a broader ecological shift. Lesional skin typically shows a significant decrease in alpha diversity, with a higher relative abundance of the phylum Firmicutes and a corresponding decrease in Actinobacteria.^110^, ^111^ Several studies report a relative increase in *Corynebacterium* (and in some cohorts *Streptococcus*) together with a marked depletion of commensal *Cutibacterium species*, including *C. acnes*.^130^, ^131^

This cutaneous dysbiosis is intrinsically linked to systemic health via the gut-skin axis (Fig. 3). Psoriatic patients often show gut dysbiosis, including reduced abundance of the anti‑inflammatory butyrate producer *Faecalibacterium prausnitzii*, and in some cohorts an increased abundance of pro‑inflammatory taxa such as *Prevotella copri*.^131^, ^132^ As illustrated in Fig. 3, the resulting “leaky gut” allows for the translocation of bacterial components (e.g., LPS) into systemic circulation. This systemic “priming” of the immune system triggers the IL-23/IL-17 signaling pathway, driving the recruitment of Th17 cells to the skin and exacerbating the inflammatory feedback loop that maintains psoriatic lesions.^133^

## Modern research frontiers and therapeutic horizons

Advances in molecular technologies have rapidly changed the field of skin microbiome research from being an observational/descriptive science to a functional and interventional science. The exploration of not just outlining microbial communities on the skin surface, but understanding the functional outputs from those communities, is now being explored at an unprecedented scale. Many new advancements in science can be defined as new frontiers and horizons, bringing about high resolution using multi-omics techniques, innovative microbiome-based interventions, perhaps even the dream goal of engineering the skin's microbial ecosystem for therapeutic effect. As this field matures toward more personalized, precise, and ecologically aware dermatological treatments (versus using simple broad-spectrum antimicrobials) will enable consideration of how to restore an established and healthy, or soon-to-be established healthy and balanced microbial community.

### High-resolution sequencing and multi-omics approaches

Much of the work in early skin microbiome research utilized culture-based methods to identify and quantify microbial populations; however, it has been ultimately unsuccessful as culture methods only identify viable organisms and completely exclude dead organisms, and narrowly identify potentially pathogenic organisms. Based on studies prior to 2000, most skin remains undiscovered by early cultures, and therefore it is not surprising that researchers have moved toward culture-independent methods to fully describe microbiomes.^134^, ^135^ Following the development of 16S rRNA gene sequencing, the authors are now seeing a move towards higher-resolution sequencing that goes beyond simple identification of microbial communities. Metagenomic sequencing (i.e., assessing the total collective genome of the microbial community) allows for species identification and could even further elaborate to strain-level identification and phylogenetic organization of the specific species identified, while also revealing the potential functional attributes encoded in the microbiome's genes.

The merging of the various “omics” fields is helping develop a more holistic ecosystem of the skin. Metatranscriptomics (gene expression), metaproteomics (protein production), and metabolomics (metabolic byproduct profiling) are telling us what the microbes are actively "doing", and how they interact with the host. Understanding the "functions" of biodiverse microbial communities will help to identify causative factors involved in skin diseases, not just demonstrating correlation.^136^ For example, these various approaches will identify microbial pathways that produce inflammatory metabolites or bacterial enzymes that degrade elements of the skin barrier. Combining high-resolution sequencing techniques with functional experiments using individually isolated microbes is proving absolutely essential to unearthing these functions and identifying new targets for intervention.^137^, ^138^

### Microbiome-centered interventions: probiotics, prebiotics, and postbiotics

The growing understanding of dysbiosis has led to the development of various interventions to stymie dysbiosis and alter the skin microbiome. In general, these interventions can be grouped into three categories: probiotics, prebiotics, and postbiotics.

• Probiotics are defined as live microorganisms that, when given in adequate amounts, confer a health benefit to the host. In dermatology, this currently means applying genetically relevant beneficial strains, like *Lactobacillus* species or non-pathobiont strains like *S. epidermidis,* on the skin to outcompete pathobionts, strengthen the skin barrier, and modulate local immune responses.^10^, ^113^, ^139^, ^140^
• Prebiotics are defined as substrates that are selectively metabolized by host microorganisms, conferring a health benefit. They are essentially “food” for beneficial microbes. Ingredients like oligosaccharides, inulin, and some plant extracts can be incorporated into topical formulations to be preferentially metabolized by commensal bacteria as a means to restore a healthy microbial balance.^120^, ^141^
• Postbiotics are metabolic byproducts from probiotic microorganisms, such as short-chain fatty acids, enzymes, and antimicrobial peptides, as well as non-viable bacterial cells or cell fractions. Their application to the skin or uses in therapeutic products achieves the health benefits of live microbes and topical probiotics while avoiding the significant challenge of maintaining viability of live organisms in cosmetic or therapeutic formulations.^142^, ^143^

**Fig. 5:**
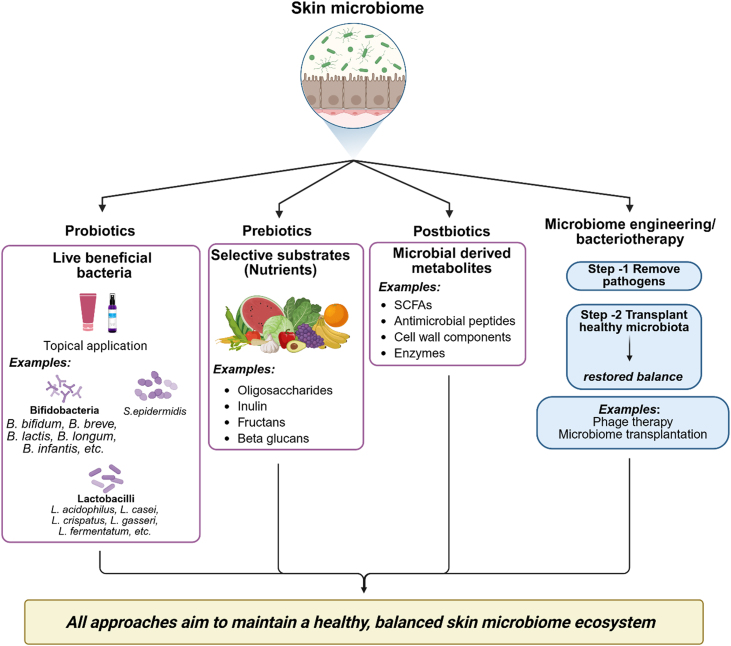
**Therapeutic strategies for skin microbiome modulation.** Overview of interventions, including probiotics (live bacteria), prebiotics (selective substrates), postbiotics (microbial metabolites), and microbiome engineering (bacteriotherapy and transplantation), aimed at restoring a balanced cutaneous ecosystem. Created in https://BioRender.com.

These “biotics” represent a significant paradigm shift in dermatological care and treatment, providing a more selective and gentle option than using harsh antimicrobials. An increasing number of studies support their utility in inflammatory conditions like AD and as an option to maintain healthy skin and skin barrier function (Fig. 5).

### Skin microbiome engineering: Challenges and opportunities

The most ambitious area of skin microbiome application, other than introducing existing microbes or their metabolites, is skin microbiome engineering, an emerging discipline that seeks to rationally and precisely alter the composition and function of skin microbial communities to prevent or heal disease.^144^, ^145^ This could involve the use of several high-risk approaches:

• Bacteriotherapy: Topical application of beneficial bacterial strains (autologous or commensal) corrects skin dysbiosis, akin to fecal microbiota transplantation for gut diseases. Pilot studies in AD showed reduced *S. aureus* and symptom improvement.^82^, ^138^, ^146^
• Synthetic communities: Researchers are working to develop and assemble defined consortia of beneficial microbes with specific and synergistic functions instead of providing whole, undefined communities.^137^, ^147^ These synthetic communities could specifically target certain dysbiotic states; for instance, re-establishing colonization resistance against *S. aureus*.
• Genetic engineering of commensals: This idea entails modifying the genome of one of the skin-resident bacteria, such as *S. epidermidis*, as a “living therapeutic”.^73^ These microorganisms could be programmed to produce skin lipids, anti-inflammatory molecules, or antimicrobial peptides on demand, directly at the disease site.

Skin microbiome engineering represents incredible potential, but challenges remain, such as ensuring the safety and stability of the engineered microorganisms, addressing complex regulatory pathways, and navigating the ethical ramifications. The evolution of skin microbiome engineering represents a distinct shift toward proactive, personalized, and curative therapies across the dermatological spectrum of skin diseases.

### Future directions in personalized dermocosmetics and therapeutics

The future of dermatology will ultimately shift toward personalization, with the microbiome as the foundation. Given the massive inter-individual variability in skin microbial communities, the one-size-fits-all paradigm to skincare and treatment is often inadequate. High throughput sequencing may become routine in the future, and clinicians will be able to characterize a patient's microbiome and define specific dysbiotic signatures.

This information would allow personalized dermocosmetic and therapeutic formulations. Imagine a day when consumers can purchase moisturizers that contain prebiotic ingredients specifically selected to boost the uniqueness of commensal populations, or when acne patients have access to topical medications including bacteriophages that selectively eradicate the culpable, pro-inflammatory strains of *C. acnes*. For chronic skin disorders, clinicians may prescribe an engineered living biotherapeutic to address a patient's unique microbiome-driven immunological or metabolic deficit. The promise of these personalized approaches would result in more effective, safer, and longer-lasting approaches to skin health and diseases, and a true merger of ecology, molecular biology, and clinical dermatology.

## Conclusion

The study of the human skin microbiome has evolved dramatically from initial microbiological cataloging to an advanced ecological science. This transformation, catalyzed by Mary Marples' pioneering work in the 1960s, established the foundational principle that skin harbors not merely contaminating bacteria, but rather a complex, functionally integrated microbial ecosystem. The evolution of skin microbiome study was greatly enhanced by culture-independent molecular techniques, which captured the skin microbiome as a community of bacteria, fungi, viruses, and mites, all part of skin health. The microbiome represents more than passive colonizers and is a participant in homeostasis in many ways, including maintaining the physical barrier of skin, educating (and modulating) the host immune system, and performing a host of metabolic processes.

However, this ecosystem can become disrupted and lead to dysbiosis, which is increasingly recognized as a possible pathogenesis of various dermatological conditions, including AD, acne vulgaris, and chronic wounds. The challenge of modern research is to expand beyond correlation-based findings and delineate the mechanistic causation of how these microbial changes create a disease state. The authors are at the precipice of a new wave of skin microbiome research characterized by high-resolution sequencing and multi-omics technologies. The goal of these investigations is to learn not only who is there, but what these total communities are doing.

The authors are beginning to understand how forward-thinking approaches can lead to new treatment paradigms. Traditional therapies that relied on antibiotics are being expanded into more intricate treatment modalities, such as the use of probiotics, prebiotics, and postbiotics to restore microbial balance through therapeutic interventions. This is only the beginning of our ability to engineer the skin microbiome, as there are still many hurdles to overcome, but the possibility for targeted and personalized treatments for chronic dermatological diseases will eventually emerge. Research will only accelerate as the authors combine the ecological principles with forefront research tools. Our use of the word “future” for skin health is an intentional reference to the microbes that inhabit our skin.

## Research data availability

The entire dataset supporting the results of this study was published in this article.

## Financial support

This study was funded by Fundação de Amparo à Pesquisa do Estado de São Paulo (10.13039/501100001807FAPESP), grant2022/09354-9).

## Author contributions

Sabri Saeed Sanabani: Approval of the final version of the manuscript; critical literature review; manuscript critical review; preparation and writing of the manuscript; study conception and planning.

## Conflicts of interest

None declared.
